# Planning and managing the physician workforce

**DOI:** 10.1186/2045-4015-1-14

**Published:** 2012-03-12

**Authors:** Stephen C Schoenbaum

**Affiliations:** 1 Josiah Macy Jr. Foundation, 44 East 64th Street, New York, NY 10065 USA

## Abstract

National planning and management of the physician workforce is a multifaceted, difficult, and even controversial activity. It is an important subset of overall health workforce planning and management, which contributes to a country's having an effective and efficient health care system. This commentary builds on a new survey of specialty considerations by Israeli medical students early in their clinical training, places it in the broader context of health workforce planning, and provides examples of some approaches and activities being taken in the United States that are applicable to other developed countries.

This is a commentary on http://www.ijhpr.org/content/1/1/13.

## Commentary

In any country the ability of persons to get the health care services they need depends upon the size, composition, and accessibility of that country's health care workforce and health care facilities. The country's supply of physicians is a subset of the overall health care workforce. In turn, the supplies of specific types of physician generalists and specialists are subdivisions of the total physician supply. Furthermore, within countries there tends to be large geographic variation in the distribution of the various elements of the health care workforce, which affects accessibility of services or certain types of services.

It appears that each of the abovementioned issues is relevant in Israel. Recently, to address an apparent shortage of domestically trained and retained physicians, medical schools have been increasing their class sizes, and a new medical school has opened for the first time since 1974. There has also been concern that new graduates of Israeli medical schools are not choosing to train in fields such as internal medicine, general surgery, and anesthesiology, and concern about the tendency of physicians to locate centrally within the country rather than in the periphery. It is in that context that Weissman et al. have surveyed fifth year medical students, studied medical specialty considerations at that stage of their training, and pointed out the opportunity to influence their specialty considerations in the early stages of clinical training [1].

Workforce assessment and planning efforts are complex. Although the issues I present below are generally applicable to developed countries, my examples come primarily from the United States. Within the U.S., workforce assessment and planning efforts are not only complex but highly controversial and sometimes politically sensitive. The controversies relate to the data sources, the methods of analysis, and whether approaches to addressing workforce issues should be through policies and regulation or market forces [2-4]. A surplus of physicians for the 2000s that was projected in the mid-1990s based on the growth at the time of managed care and the way managed care organizations used physicians and other health professionals [5], failed to materialize [3]. An increased choice of primary care training by new physicians in the late 1990s, probably driven by the prevalence at the time of managed care and its use of primary care gatekeepers, not only failed to be sustained but reversed dramatically and is now a significant issue [6]. There is also controversy about the degree to which the U.S. will have a future shortage of specialists [7]. And there is controversy about the relationship of care outcomes to physician supply [8]. It is argued that a major factor in care outcomes is primary care physician supply [9]. Indeed, there is evidence from around the world that more primary care services, not necessarily delivered solely by physicians but also by nurses and other health professionals, are associated with better outcomes, lower costs, and greater equity of care [10].

Turning to the seemingly more circumscribed issue of medical student choices of specialties, it is well known that multiple factors are involved [6,11-13]. The article by Weissman et al. [1] becomes important in assessing some of these factors if Israel's policy-makers believe there are problems that need addressing. If so, it is not merely an article about the considerations of current fifth year medical students but a starting point for determining whether and how those considerations might be altered to best serve the country's needs. Weissman et al. point to opportunities "to address the shortages of physicians in certain specialties", including the "design of selection processes, medical school curriculum experiences, role model exposure, career counseling services and incentives..." Each of these merits attention.

The U.S. is not known for central planning or management of its health care workforce or even its physician workforce. Indeed, like most other aspects of health care in the U.S., workforce planning and management, to the extent it occurs, has been highly fragmented. As stated by The Robert Graham Center in Washington, DC, "Unlike many Western nations, the United States does not manage or actively regulate the number, type, or geographic distribution of its physician workforce." [11] That said, there have been frequent national efforts to assess and make recommendations about the physician workforce. The U.S. Congress in 1986 authorized a Council on Graduate Medical Education (COGME) "... to provide an ongoing assessment of physician workforce trends, training issues, and financing policies and to recommend appropriate Federal and private-sector efforts to address identified needs." [14] The U.S.'s 2010 health reform legislation [15,16] included a provision that established a 15-person National Health Care Workforce Commission. Although persons were appointed to the Commission, it subsequently was not funded by Congress and has not become active. There is, however, an operational National Center for Health Workforce Analysis within the U.S. Department of Health and Human Service's Health Resources and Services Administration.

It is now recognized that a very large percentage of Americans have one or more chronic conditions and that management of persons with multiple chronic conditions accounts for a disproportionate share of the U.S.'s very high levels of health spending. Those conditions are managed primarily in ambulatory settings. Persons from several developed countries with those conditions report frequently having problems related to fragmented care; and they appear to benefit from having a primary care "medical home" to coordinate their care [17]. This line of reasoning has led to several provisions within the U.S. health reform legislation designed to expand access to primary care and provision of primary care services [18]. These include workforce training provisions such as expanded student loan programs in primary care and nursing. In addition, concern about the supply of primary care physicians in the U.S. and the degree to which graduate medical education is providing an appropriate primary care workforce has led to recent recommendations by COGME [14], recommendations from other groups [19], and a variety of efforts to address several of the factors raised by Weissman et al. [1] that are involved in physician choice of specialties.

A few of these efforts are worth mentioning here: First, new medical schools, both allopathic and osteopathic, have been opening in the U.S. for over a decade. In part this has been a response to the fact that for many years U.S. medical schools have not produced enough physicians to fill graduate medical training positions. Indeed, for many years, about one-quarter of all U.S. residency slots have been filled by international medical graduates (IMGs), particularly in fields such as family medicine, internal medicine, and psychiatry. Osteopathic medical school graduates have been more likely to enter primary care than are allopathic medical school graduates, and some of the new allopathic medical schools have been adopting new educational models in hopes of training physicians who have a better grounding in ambulatory care and are more likely to choose generalist fields [20]. Role model exposure is related to the "hidden curriculum", the lessons that medical students learn from those around them and not just from the formal curriculum. It is also related to faculty development efforts [21], to evaluation of faculty for providing appropriate role modeling, and to professionalism. Efforts to formally integrate education about professionalism into medical education curricula are ongoing in most if not all U.S. medical schools.

As U.S. students are thinking about and deciding on their choice of medical specialty, it is known that they seriously consider their future income [11]. I believe that not just in the U.S., students also consider what their actual daily work will be like once they have trained. In part, that involves important lifestyle issues - e.g., ability to work scheduled hours or part-time, and amount of on-call responsibility. In part, it should involve understanding the essential roles for which physicians trained in various fields will be needed. I think that for both specialists and generalists, too little attention has been paid to defining these essential roles. In the U.S. some of the roles are changing and are likely to continue to change. One possible change is the degree to which physicians, vs. other members of a team, such as highly trained technicians, will be performing some technical aspects of specialty care. Another is the degree to which delivering primary care will be the responsibility of physicians vs. other professionals, e.g., nurse practitioners, physicians' assistants. It is now recognized that in the twenty-first century, teams of health professionals will be needed to provide care, not just in settings such as operating rooms, but also for patients needing post-acute and chronic care. Physicians and other health professionals will need to know how teams can be organized and function most effectively. They will need to know what they can best contribute to the team and what can most effectively be contributed by others.

Interestingly, Weissman et al. [1] found that only about a third of the surveyed Israeli medical students were interested in specialties with much teamwork. It is known that team care can be facilitated by interprofessional education (IPE). IPE has been defined by the World Health Organization as "... when students from two or more professions learn about, from and with each other to enable effective collaboration and improve health outcomes." [22] Teamwork can also be facilitated by enabling different health professional groups "... to practice to the full extent of their education and training", a formal recommendation of the U.S. Institute of Medicine with respect to nursing [23], which can be applied to all health professionals. Indeed just among physicians, to achieve more efficient and effective practice, it will be important for specialists and subspecialists to teach generalists knowledge and skills that need not be the exclusive domain of the more specialized physicians.

Finally, as mentioned by Weissman et al. [1], incentives can play a significant role in influencing physician choice of specialty. These can include the length of training, availability of training positions, financial rewards, and possibly others. In the U.S., at Texas Tech, there is new curriculum for students committing to a career in family medicine that enables medical school graduation in three vs. four years (post-college). U.S. health reform legislation includes some direct financial rewards for primary care practitioners, stimulates development of enhanced primary care practices or medical homes, provides bonuses for primary care physicians who practice in underserved areas, and for physicians in rural areas to be paid as much as those in urban areas [18].

In short, there are many ways to influence physician choice of specialty--ensuring that the physician workforce has the right composition is an important piece, but hardly the only important piece, of health workforce planning. One might wonder if there is a blueprint to guide health workforce planning so that it is done right. The answer appears to be that there is no single way to approach the task. But fortunately, there are instructive case examples of different national approaches to health workforce planning and management [24-26]. As with providing universal coverage for health care services [27], each country is likely to need to take an approach that grows from understanding the specific problems that need to be addressed and, importantly, from national values related to workforce and healthcare. Both Israel and the U.S. need to consider these elements and then determine the structures, policies, and practices they need to implement or enhance to attain their desired goals.

## Competing interests

The author declares that he has no competing interests.

## Author's information

Stephen C. Schoenbaum, MD, MPH, is Special Advisor to the President of the Josiah Macy Jr. Foundation. From 2000 to 2010, he was Executive Vice President for Programs at The Commonwealth Fund and Executive Director of its Commission on a High Performance Health System. He has had extensive experience as a clinician, epidemiologist, and manager.
